# Epitope Mapping of Antibodies to Alpha-Synuclein in LRRK2 Mutation Carriers, Idiopathic Parkinson Disease Patients, and Healthy Controls

**DOI:** 10.3389/fnagi.2014.00169

**Published:** 2014-07-15

**Authors:** Beatriz Alvarez-Castelao, Ana Gorostidi, Javier Ruíz-Martínez, Adolfo López de Munain, José G. Castaño

**Affiliations:** ^1^Departamento de Bioquímica, Instituto de Investigaciones Biomédicas “Alberto Sols”, UAM-CSIC, Facultad de Medicina, Universidad Autónoma de Madrid, Madrid, Spain; ^2^Centro de Investigación Biomédica en Red sobre Enfermedades Neurodegenerativas (CIBERNED), Madrid, Spain; ^3^Servicio de Neurología, Hospital Donostia, San Sebastián, Spain; ^4^Area de Neurociencias, Instituto Biodonostia, San Sebastián, Spain; ^5^Departamento de Neurociencias, Universidad del País Vasco-Euskal Herriko Unibertsitatea, San Sebastián, Spain

**Keywords:** alpha-synuclein, human antibodies, ELISA, immunoblot, epitope mapping, LRRK2 mutation, Parkinson disease

## Abstract

Alpha-synuclein (Snca) plays a major role in Parkinson disease (PD). Circulating anti-Snca antibodies has been described in PD patients and healthy controls, but they have been poorly characterized. This study was designed to assess the prevalence of anti-Snca reactivity in human subjects carrying the LRRK2 mutation, idiopathic PD (iPD) patients, and healthy controls and to map the epitopes of the anti-Snca antibodies. Antibodies to Snca were detected by ELISA and immunoblotting using purified recombinant Snca in plasma from individuals carrying LRRK2 mutations (104), iPD patients (59), and healthy controls (83). Epitopes of antibodies were mapped using recombinant protein constructs comprising different regions of Snca. Clear positive anti-Snca reactivity showed no correlation with age, sex, years of evolution, or the disability scores for PD patients and anti-Snca reactivity was not prevalent in human patients with other neurological or autoimmune diseases. Thirteen of the positive individuals were carriers of LRRK2 mutations either non-manifesting (8 out 49 screened) or manifesting (5 positive out 55), three positive (out of 59) were iPD patients, and five positive (out of 83) were healthy controls. Epitope mapping showed that antibodies against the N-terminal (a.a. 1–60) or C-terminal (a.a. 109–140) regions of Snca predominate in LRRK2 mutation carriers and iPD patients, being N122 a critical amino acid for recognition by the anti-C-terminal directed antibodies. Anti-Snca circulating antibodies seem to cluster within families carrying the LRRK2 mutation indicating possible genetic or common environmental factors in the generation of anti-Snca antibodies. These results suggest that case-controls’ studies are insufficient and further studies in family cohorts of patients and healthy controls should be undertaken, to progress in the understanding of the possible relationship of anti-Snca antibodies and PD pathology.

## Introduction

Parkinson disease (PD) and other synucleinopathies (dementia with Lewy bodies and multiple system atrophy) are characterized pathologically by proteinaceous inclusions commonly described as Lewy pathology in postmortem brain tissue samples (Goedert, 2001). Alpha-synuclein (Snca) is the major protein constituent of Lewy bodies (Spillantini et al., 1998). Snca is mainly a cytoplasmic protein, but it has been shown to be secreted by exocytosis and is also present in exosomes (Lee et al., 2005; Emmanouilidou et al., 2010; Jang et al., 2010; Pan-Montojo et al., 2012). Snca has been found in cerebrospinal fluid (CSF) and plasma and its concentration in these fluids has been suggested as a possible biomarker for PD (Parnetti et al., 2013), even though there are substantial discrepancies between the results reported in different studies (Lee et al., 2006; Li et al., 2007; Duran et al., 2010; Shi et al., 2010; Mollenhauer et al., 2011; Park et al., 2011; Foulds et al., 2012; Wennstrom et al., 2013). In this context, recent measurements of clearance of Snca in CSF under steady-state conditions in PD patients and PD animal models show that there is no change in the clearance of Snca in the CSF respect to the corresponding controls (Fanara et al., 2012). Further interest in extracellular Snca is due to data showing that extracellular Snca in an aggregated form may have a potential role in the spreading of the PD pathology, as suggested by the finding of Lewy bodies in fetal neurons transplanted to PD patients (Kordower et al., 2008; Li et al., 2008; Mendez et al., 2008) and the spreading of aggregation of neuronal endogenous Snca in response to the addition (cells) or injection (animals) of aggregated fibrillar Snca (Luk et al., 2009, 2012a,b; Volpicelli-Daley et al., 2011). The extracellular behavior of Snca is also part of the rationale for an immunological intervention as possible treatment for PD. Active (Masliah et al., 2005) or passive immunization (Masliah et al., 2011) against Snca has been shown to be effective in prevention of the pathology observed in mice models overexpressing human Snca.

The study of anti-Snca antibodies, as a possible biomarker for PD, has also received considerable attention. The initial study of the presence of antibodies against Snca in human subjects was undertaken because commercially available anti-latent protein 1 EBV antibodies cross-reacted with Snca (Woulfe et al., 2000) and later the same authors reported no difference in prevalence of anti-Snca reactivity between idiopathic PD (iPD) patients and healthy controls (Woulfe et al., 2002). Afterwards, multi-epitopic Snca antibodies were found in patients from families with uncharacterized familial forms of PD, but its frequency was not significantly higher in iPD patients with respect to healthy controls (Papachroni et al., 2007). Later studies have shown that there is no significant difference in the presence of anti-Snca antibodies between iPD patients and healthy controls (Smith et al., 2012; Besong-Agbo et al., 2013), while other studies found significant differences, with higher frequency in iPD patients (Yanamandra et al., 2011). Nevertheless, the human anti-Snca antibodies have been poorly characterized.

The present study was designed to evaluate the presence of antibodies against Snca in subjects with dominant LRRK2 mutations, asymptomatic (non-manifesting carriers) and symptomatic (manifesting carriers), iPD patients (manifesting non-carriers), and healthy controls, all of them reside in the Basque country (Spain). We took advantage of the fact that LRRK2 mutation, a well known cause of familial PD, is responsible for 46% of familial PD and for 2.5% of sporadic PD in the PD population of Basque ascent (Ruiz-Martinez et al., 2010). Sera form patients with other neurological or autoimmune diseases were also evaluated for the presence of anti-Snca antibodies, as a way to study disease specificity. Finally, we also report the mapping of the major epitopes of the anti-Snca antibodies present in non-manifesting and manifesting LRRK2 carriers, iPD patients, and healthy controls.

## Materials and Methods

### Patients

Plasma from 49 asymptomatic (non-manifesting carriers) LRRK2 mutation carriers (Asymp LRRK2) and 55 symptomatic (manifesting carriers) LRRK2 mutation carriers (Symp LRRK2 PD) belong to 38 different families, 55 iPD patients (manifesting non-carriers), and 83 healthy controls were all recruited from the Movement Disorders Unit (MDUD) of the Hospital Universitario Donostia, San Sebastian, Spain. All iPD patients and healthy controls were negative for LRRK2 mutations. These patients and controls were mainly from the Basque region of Spain, where the prevalence of LRRK2 mutation is significantly higher than in other Spanish regions. Information recorded for each subject included age, gender, time of symptoms onset, and date of first diagnosis. Patients with PD fulfilled the diagnostic criteria of PD (Gelb et al., 1999) and Parkinson’s disability scores using Unified Parkinson’s Disease Rating Scale (UPDRS-III) (Goetz et al., 2007) and the Hoehn and Yahr (H&Y) scale (Hoehn and Yahr, 2001) were also measured. Demographic data of patients and healthy controls are summarized in Table 1. We also analyzed sera from 17 patients with progressive supranuclear palsy (PSP) that were residents in the Basque country, 43 patients with Alzheimer’s disease (AD) provided by Dr. Ana Frank, IdiPaz, Department of Neurology, La Paz University Hospital, Madrid, Spain, 45 patients with diagnosis of relapsing–remitting MS derived from our own previous studies (Mayo et al., 2002) and their demographic data are summarized in Table S1 in Supplementary Material. Table S1 in Supplementary Material also presents the demographical data of other patients whose sera were also screened for anti-Snca antibodies: 36 patients with diagnosis of primary biliary cirrhosis (PBC), 25 patients with diagnosis of systemic lupus erythematosus (SLE), 15 patients with diagnosis of Sjögren syndrome, and 39 healthy controls. Patients and healthy controls summarized in Table S1 in Supplementary Material were resident in the area of Madrid, Spain and were provided by Dr. Rita Alvarez Do-Forno, IdiPaz, Department of Immunology, La Paz University Hospital, Madrid. The current investigation was approved by the Ethical Committee of the participating hospitals and conducted according to the Declaration of Helsinki principles and informed consent was obtained from all subjects.

**Table 1:** **Demographical characteristics of patients and healthy control subjects used for the screening of antibodies against Snca**.

Characteristics	Condition			
	Asymptomatic LRRK2 carriers	Symptomatic LRRK2 carriers	Idiopathic PD	Healthy control subjects
Age (mean ± SD)	51.18 ± 11.3	68.37 ± 10.23	67.81 ± 9.98	61.4 ± 14.7
Sex (M/F)	23/26	23/32	34/25	38/45
Number of patients with LRRK2 mutation R1441G/G2019S	43/6	39/16	NA	NA
Age onset	NA	61 ± 9	62 ± 12	NA
Years evolution up to date of testing (mean ± SD)	NA	13 ± 11	12 ± 8.7	NA
UDPRS (mean ± SD)	NA	26 ± 13	28 ± 12	NA
Hoehn and Yahr score (mean ± SD)	NA	2.25 ± 0.88	2.44 ± 0.80	NA
Clear-cut positive anti-Snca/total screened (OD ≥ 0.5 by endpoint ELISA and titer by immunoblot ≥1/200)	8/49	5/55	3/59	5/83

*NA, not applied*.

### Cloning, bacterial expression, and purification of recombinant proteins

Recombinant wild type (wt) Snca and the point mutants, A30P, E46K, and A53T were purified as described (Martin-Clemente et al., 2004; Alvarez-Castelao and Castano, 2011). Because of the results obtained with this initial preparation, we need to add one further step of purification of the recombinant Snca by RP-HPLC on a Vydac-C18 column equilibrated in 0.1% TFA and elution with a linear gradient (0–40% acetonitrile in 0.1% TFA). The fractions containing Snca were pooled lyophilized and store frozen at −70°C until used. Deletion constructs, Snca stop109, His-tag Snca, His-tag 1–60, and His-tag 61–140 hSnca wt, were purified from induced BL-21 bacterial cultures as described (Alvarez-Castelao and Castano, 2011; Alvarez-Castelao et al., 2014).

### ELISA and Western immunoblot analysis

To detect anti-Snca antibodies, 100 μL of recombinant Snca (10 μg/mL) in 0.1 M Na_2_CO_3_ was coated overnight on 96-well ELISA microtitration plates (Maxisorp; Nunc, Roskilde, Denmark). After three washes with 250 μL of TTBS (0.1% Tween 20 in 1×TBS: 50 mM Tris–Cl pH 7.4, 150 mM NaCl), the wells were blocked with 150 μL of TTBS containing 3% BSA (Sigma A3803) and incubated for 2 h at 37°C to avoid non-specific binding. After three washes with 250 μL of TTBS, the plasma or serum samples (100 μL, starting from a 1:100 dilution in TTBS with 1% BSA) were placed into the wells and incubated for 60 min at room temperature (RT). After four washes with 250 μL of TTBS, 100 μL of secondary antibody goat anti-human IgG (Sigma, 1:2000) diluted in TTBS + 1% BSA was added to each well and incubated for 1 h at RT. Wells were washed four times with TTBS and 100 μL of developing reagent containing *p*-nitrophenyl-phosphate was added. Plates were incubated at RT and kinetic readings were obtained in a microplate reader (Infinity M1000, Tecan) at 405 nm. End point values were obtained after 1 h incubation at RT. Accuracy of the ELISA assay was determined by 1:2 dilutions with a negative sample of plasma/sera from five different patients with different levels of reactivity. Intra-assay precision was determined by testing 10 replicates of each (low, medium, and high) range of reactivity and the CV calculated for each of the concentration range from their respective average and SD. Inter-assay precision was determined by testing three (1:100) dilutions of each (low, medium and high) reactivity range and testing at two different occasions, 1 week apart, the CV was calculated from their respective average and SD. No difference was found by using sera or plasma in the detection of anti-Snca antibodies.

For immunoblot analysis, 1–2 μg of purified recombinant proteins were separated on 14 or 16% SDS-PAGE. Gels were transferred to nitrocellulose or PVDF for immunoblotting. Membranes were blocked overnight at 4°C with blocking buffer TTBS with 3% BSA. The membranes were incubated for 2 h at RT with plasma or sera from human subjects (unless otherwise indicated) at 1:100 dilution. After washing three times with TTBS (10 min each), the blots were incubated with alkaline phosphatase labeled goat anti-human IgG antibodies (Sigma) at 1:2000 dilution for 1 h at RT, washed three times (10 min each) with TTBS and developed with nitro-blue tetrazolium and 5-bromo-4-chloro-3′-indolyphosphate, as described (Mayo et al., 2002).

### Antibody-affinity purification

Purified recombinant Snca was subjected to continuous 14% SDS-PAGE and western blotted. The horizontal strip containing Snca was excised, blocked with TTBS containing 3% BSA, and incubated for 3 h at RT with 0.4 mL of patient serum prepared in the same buffer. The membrane was then washed with TTBS three times (10 min each) and the bound antibodies were eluted by incubation with 0.2 M Gly, pH 2.4. The solution containing the eluted antibodies was immediately neutralized with 1 M Tris–Cl pH 7.4 and diluted threefold with TTBS. Finally, the neutralized and diluted solution was used for further analysis.

### Mice samples

Snca (−/−) knock-out mice, their corresponding control wt mice (+/+), and transgenic mice for human wt Snca under the control of the tyrosine hydroxylase (TH) promoter were provided by Dr. Isabel Fariñas, Facultad de Biología, Universidad de Valencia and have been described previously (Abeliovich et al., 2000; Perez-Sanchez et al., 2010). Animals were housed and sacrificed following the local legislation in agreement with the European Union guidelines (86/609/EEC and 2003/65/EC, European Council Directives). Mice were anesthetized and whole brains were removed, fresh brains were subjected to dissection, cut into coronal sections and immediately frozen in liquid nitrogen and stored at −80°C until further processing. Crude extracts were obtained by homogenization of the brain sections in TTBS (1/4 weight/volume) and centrifuged at 14,000×*g* for 30 min at 4°C to remove insoluble materials. The extracts were loaded (50 μg of total protein) onto 14% SDS-PAGE, western blotted, and processed for immunoblotting as described above. Anti-tubulin (Sigma) antibodies were used as control of protein loading.

### Statistical methods

Statistical analysis was performed using the SPSS 15.0 software package (SPSS Inc., Chicago, IL, USA). The statistical difference in Snca antibodies (ELISA endpoint OD readings) between different groups of patients and healthy controls was evaluated by Mann–Whitney *U*-test. The prevalence of positivity between patients groups and healthy controls was evaluated by Pearson’s chi-squared test (χ^2^).

## Results

### ELISA and immunoblot analysis of antibodies against Snca

**Figure 1:**
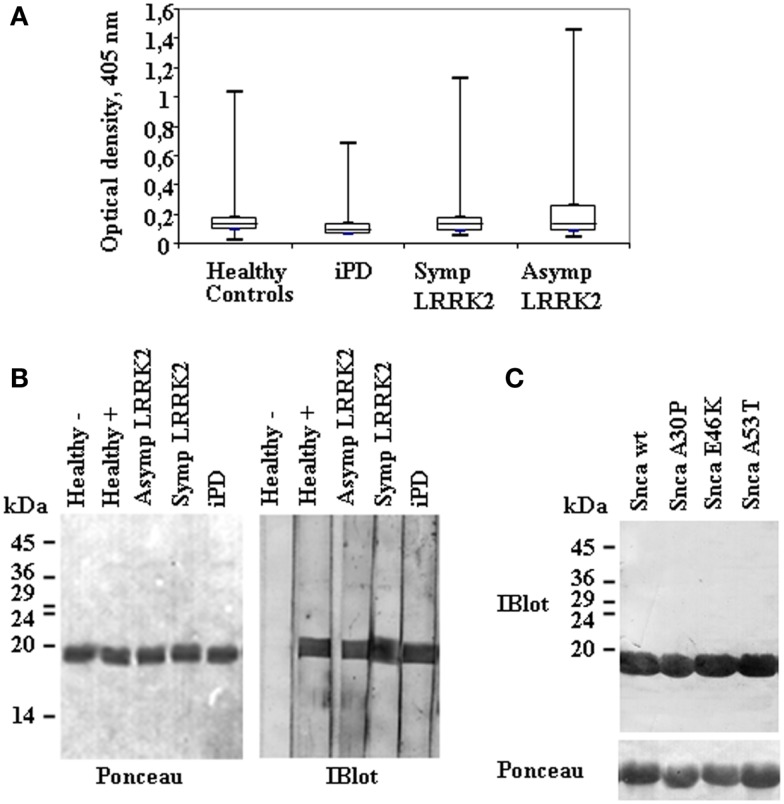
**Anti-alpha-synuclein IgG antibodies levels and representative immunoblot analysis of Snca reactivity in patients and healthy controls**. **(A)** A box plot of the results of endpoint readings of ELISA assays for antibodies against alpha-synuclein (Snca). Symbols are as follows: the median, line through the box; upper (q3) and lower (q1) quartiles, upper and lower borders of box, respectively; most extreme non-outlier values, vertical lines; and outliers, maximum and minimum values are represented by dashes for each group of individuals; healthy controls, none manifesting (Asymp) and manifesting (Symp) carriers of the LRRK2 mutation, and idiopathic PD (iPD), as indicated. Differences were not significant by Mann–Whitney *U*-test. **(B)** Continuous gels were loaded with purified recombinant Snca and western blotted; and membranes were cut into vertical strips (Ponceau staining) for testing several samples. Individual strips were incubated with plasma at 1/100 dilution. The images show representative results obtained with samples from healthy controls, negative (−) or positive (+), Asymp and Symp carriers of the LRRK2 mutation, and iPD patients that were all positive for the presence of anti-Snca antibodies. **(C)** An example of a positive sample tested against purified recombinant Snca wild type (wt) and the missense mutants A30P, E46K, and A53T.

Forty-nine non-manifesting LRRK2 mutation carriers (Asymp LRRK2), 55 manifesting LRRK2 mutation carriers (Symp LRRK2), 59 idiopathic iPD patients, and 83 healthy controls were included in the initial screening for anti-Snca antibodies, their demographic data are summarized in Table 1. The presence of Snca antibodies was determined by ELISA using purified recombinant Snca obtained after RP-HPLC purification step. For ELISA validation, accuracy and intra- and inter-assay precision tests were performed. The accuracy of the assay was determined by 1:2 dilutions of plasma/sera from five different patients and a negative sample [end point OD 0.12 ± 0.05 (SD), *n* = 30 independent assays)] that was not significantly different from the background reading obtained without addition of primary antibodies. The expected values were estimated as half of the values obtained with the undiluted sample, accuracy was then calculated as percent (expected/obtained values × 100), and the results are summarized in Table S2 in Supplementary Material. The intra-assay precision (within-run) was determined by repeating 10 times the assay of samples from patients with different levels of reactivity and the calculated CV values are presented in Table S3 in Supplementary Material. Finally, the inter-assay precision was determined by triplicate analysis of samples with different levels of reactivity in two different occasions, 1 week apart, and the results are presented in Table S4 in Supplementary Material. The results obtained validated the ELISA method used for the determination of the presence of Snca antibodies, as we obtained a good recovery (92–108%) indicating that the assay was accurate and with a good intra- and inter-assay reproducibility (CV < 15%) indicating a good precision. Endpoint ELISA titers were estimated by serial dilutions and determined as the highest dilution, which gave an OD endpoint reading >0.25 OD units, the titers obtained ranged from 1/100 to 1/1000. Comparison of endpoint ELISA OD readings (Figure 1A) of the four groups under study (patients and healthy controls) by Mann–Whitney *U*-test showed that the differences were not significant. Furthermore, no correlation was found between Snca reactivity and either age, sex, age of onset, years of disease evolution, the H&Y score, or the UPDRS.

Analysis of the Snca reactivity by immunoblot was also performed, titers by immunoblot also ranged from 1/100 to 1/1000. From the combined methods of antibody detection (ELISA and Immunoblot), we could assign “clear-cut” presence of antibodies against Snca (OD ≥ 0.5 by endpoint ELISA and titer by immunoblot ≥1/200) in 8 out 49 non-manifesting LRRK2 carriers, 5 out 55 manifesting LRRK2 carriers, 3 out 59 iPD patients, and 5 out 83 healthy controls. Examples of reactivity by immunoblot analysis are presented in Figure 1B. All those individuals that were positive against wt Snca also recognized A30P, E46K, and A53T Snca missense point mutants; an example is shown in Figure 1C. Considering only those “clear-cut” set of positive individuals, the prevalence of antibodies against Snca was higher in non-manifesting LRRK2 carriers (Asymp LRRK2) than in healthy controls (χ^2^ = 3.683, *p* = 0.05) or than in iPD patients (χ^2^ = 3.698, *p* = 0.05). All other possible comparisons (Asymp LRRK2 vs. Symp LRRK2 patients, Symp LRRK2 PD respect to iPD or healthy controls and iPD patients respect to healthy controls) were not significantly different.

To determine disease specificity, sera from patients with other neurological and autoimmune diseases were screened for the presence of anti-Snca antibodies by immunoblot analysis at 1/200 dilution. The prevalence of Snca antibodies was rare (Table S1 in Supplementary Material) in samples from patients with clinical diagnosis of AD, PSP, MS, PBC, SLE, and Sjögren syndrome. Sera of healthy controls from a different geographical region (Madrid, Spain) were also screened, because we were surprised with the number of “clear-cut” positive individuals found in the healthy control group from the Basque country and the results obtained (1 positive out 39, Table S1 in Supplementary Material) did not differ significantly respect to the healthy controls obtained from the Basque country. These results indicate that “clear-cut” anti-Snca reactivity is not very prevalent and seems not to be associated with other neurological or autoimmune diseases.

### Epitope mapping of anti-Snca antibodies

**Figure 2:**
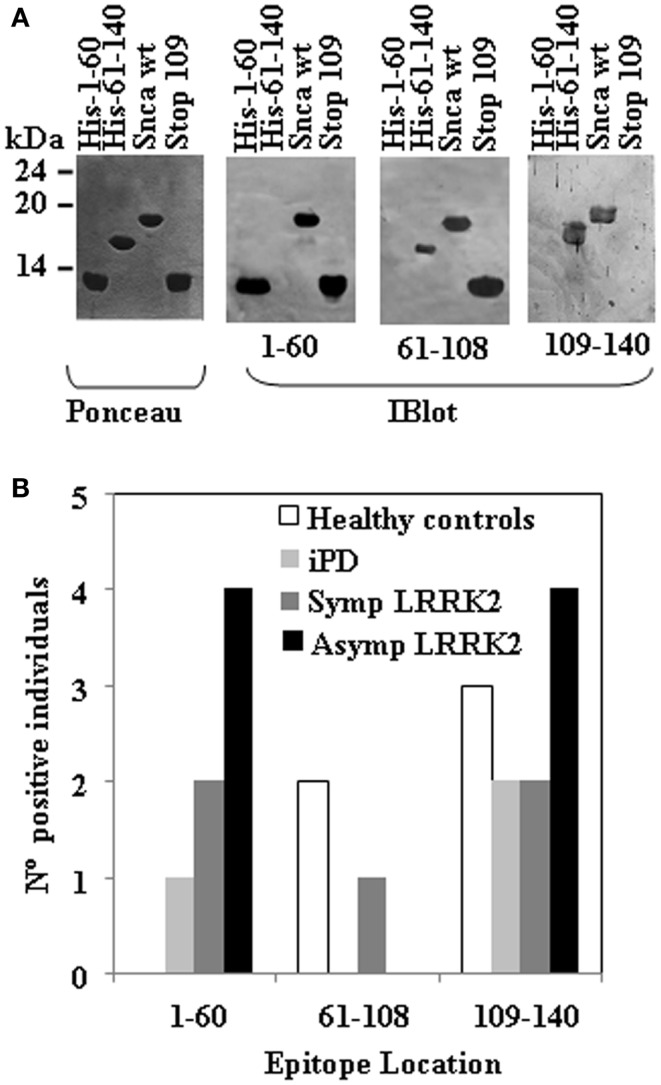
**Epitope mapping of anti-Snca human antibodies**. **(A)** The blots of the different purified Snca constructs (Ponceau) used to characterize the epitopes of anti-Snca antibodies and examples of the different types of results obtained. Plasma that contained antibodies that recognize His-Snca 1–60, Snca full length and Snca 1–108 (Stop 109), but not His-Snca 61–140 and accordingly, their main epitope must be located at the N-terminus of Snca (a.a. 1–60). Plasma that contained antibodies that recognize His-Snca 61–140, Snca full length, but not His-Snca 1–60 and Snca 1–108 (Stop 109) and as a consequence, their epitope must be located at the C-terminal region of Snca (a.a. 109–140). Finally, plasma that contained antibodies that recognize His-Snca 61–140, Snca full length and Snca 1–108 (Stop 109), but not His-Snca 1–60 and accordingly their main epitope must be located at the central region of Snca (a.a 61–108). **(B)** Graph showing the number of individuals scoring positive for the indicated regions of Snca that were found within healthy controls, iPD, and manifesting LRRK2 carriers (SympLRRK2) and non-manifesting LRRK2 carriers (Asymp LRRK2).

Next step was to map the epitopes of the human anti-Snca antibodies using different recombinant Snca constructs by western immunoblotting. To that end, we subcloned and expressed in bacteria three fragments of Snca corresponding to the N-terminal region (His-Snca a.a. 1–60), to the C-terminal region (His-Snca a.a. 61–140), and a Snca with a stop codon at position 109 (Snca a.a. 1–108). Those recombinant proteins were purified and tested together with Snca full length to map the epitopes of the human anti-Snca antibodies; the results obtained are exemplified in Figure 2A. Seven out of the 21 positive individuals had anti-Snca antibodies whose main epitope must be located at the N-terminal region of Snca (a.a. 1–60), as they reacted with protein constructs Snca 1–60 and 1–108, but not with Snca 61–140. Three out those 21 had antibodies whose main epitope must be located in the central region of Snca between positions 61 and 108, as they reacted with Snca constructs 1–108 and 61–140, but not with Snca 1–60. Finally, 11 of those 21 positive individuals had antibodies whose main epitope was located at the C-terminal region of Snca (a.a. 109–140) only reacting with protein construct 61–140, but not with Snca 1–60 or 1–108. The frequency of positive reactivity for each of the Snca protein regions as defined above is presented in Figure 2B and summarized in Table 2. There was no apparent epitope specificity that could discriminate between the four groups of individuals under analysis. Nevertheless there are clear tendencies, N-terminal (1–60) and C-terminal (109–140) specific antibodies (six times more frequent than anti-central Snca region antibodies, Table 2) were present mostly in carriers of the LRRK2 mutation (either manifesting or non-manifesting) and iPD patients, while antibodies against the N-terminal region (a.a. 1–60) were not found within positive healthy controls. Antibodies directed against the central region of Snca (a.a. 61–108) were only found in one manifesting LRRK2 carrier and in two healthy controls. The anti-Snca positive individuals carrying the LRRK2 mutation were analyzed for possible clustering in certain families (we have samples from a total of 38 different families). Family 1 with 21 members of the family screened, gave five positive individuals, four non-manifesting, and one manifesting LRRK2 carriers, and the epitopes recognized were either located at the N-terminal (a.a. 1–60) or C-terminal (109–140) regions of Snca (Table 2). Family 4 with 5 members of the family screened gave two positive, with epitopes again located at the N-terminal or C-terminal regions of Snca (Table 2). The rest of positive individuals were from different families, one out of a total of six screened from family 3, one out of two members of family 6, one out of six of family 13, one out of two of family 4, and one out one of family 20.

**Table 2:** **Epitopes of positive anti-Snca antibodies**.

Condition	Epitope 1–60	Epitope 61–108	Epitope 109–140
	
	No. positive subjects, family ID No. (mutation)	No. positive subjects, family ID No. (mutation)	No. positive subjects, family ID No. (mutation)
Asymptomatic LRRK2 carriers	2, Fam. 1 (R1441G)	0	2, Fam. 1 (R1441G)
	1, Fam. 3 (G2019S)		1, Fam. 4 (R1441G)
	1, Fam. 4 (R1441G)		1, Fam. 6 (R1441G)
Symptomatic LRRK2 carriers	1, Fam. 14 (R1441G)	1, Fam. 8 (R1441G)	1, Fam. 1 (R1441G)
	1, Fam. 20 (R1441G)		1, Fam. 13 (R1441G)
Idiopathic PD	1 (NA)	0	2 (NA)
Healthy controls	0 (NA)	2 (NA)	3 (NA)
% Positive/total positive	33.3	14.3	52.4
% Familial LRRK2 positive/total positive	28.6	4.7	28.6
% iPD positive/total positive	4.7	0	9.5
% Positive healthy controls/total positive	0	9.5	14.3

*NA, not applied*.

**Figure 3:**
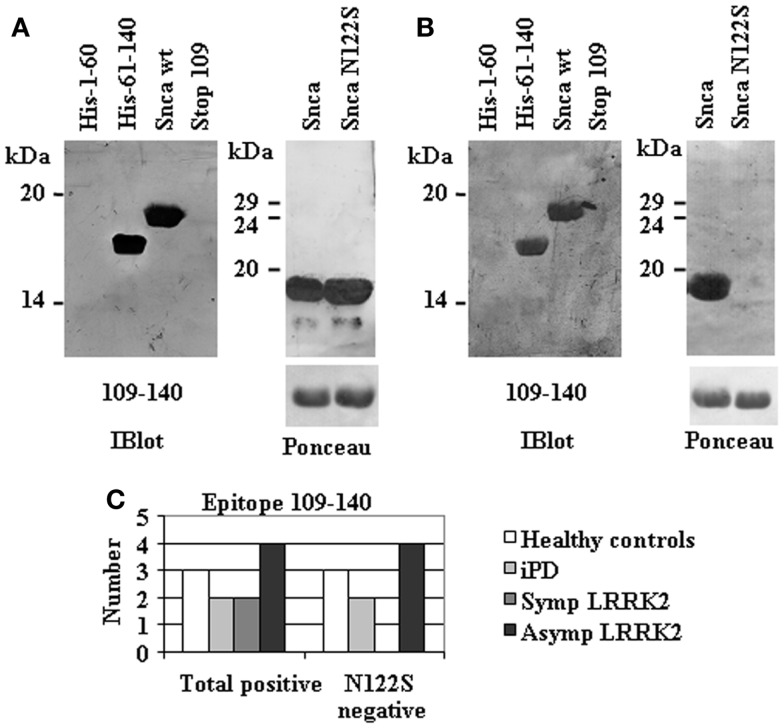
**Fine mapping of human anti-Snca antibodies directed against the C-terminal region of Snca (a.a. 109–140)**. **(A)** An example of the reactivity of human anti-Snca antibodies directed against the C-terminal region of Snca (a.a. 61–140) that also recognized Snca full length and the single point mutant Snca N122S. **(B)** An example of the reactivity of human anti-Snca antibodies directed against the C-terminal region of Snca (a.a. 61–140) that recognized Snca full length, but not the single point mutant Snca N122S. **(C)** Graph showing the total number of individuals scoring positive for antibodies against the C-terminal region of Snca and those that were negative for the N122S missense mutant within the different groups under study: healthy controls, iPD, and manifesting LRRK2 carriers (SympLRRK2) and non-manifesting LRRK2 carriers (Asymp LRRK2).

A more refined epitope mapping was obtained for those human anti-Snca antibodies directed against the C-terminal region of Snca (a.a. 109–140) by the serendipitous isolation of a Snca mutant that harbors a single missense mutation N122S obtained as an artifact of a PCR amplification. All individuals with anti-Snca antibodies whose epitope was not located at the C-terminus recognize both Snca wt and the full-length missense N122S mutant (data not shown). In contrast, two types of circulating antibodies were found in the 11 individuals whose main epitope was located at the C-terminus (a.a. 109–140). Two individuals had antibodies that recognized both Snca wt and N122S (Figure 3A), In contrast, nine individuals have antibodies that recognize Snca wt, but not Snca N122S (Figure 3B). These results indicated that amino acids around N122 constitute a major determinant for recognition by circulating antibodies recognizing the C-terminal region of Snca.

### Affinity-purified human anti-Snca antibodies

**Figure 4:**
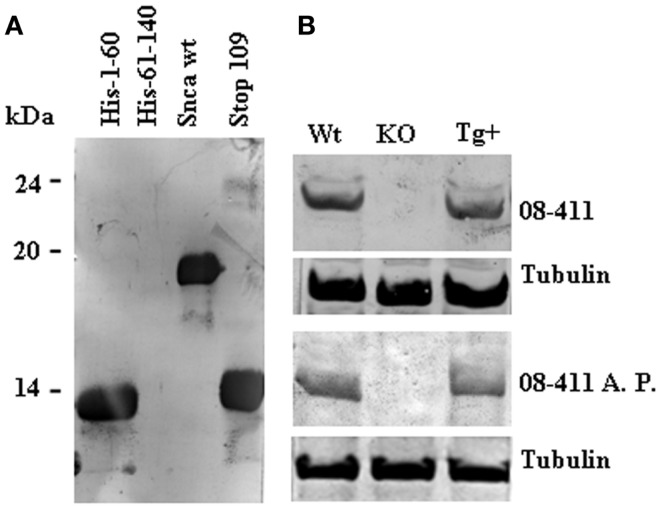
**Immunoreactivity of human anti-Snca antibodies against brain extracts from wild type (wt), Snca knock-out, and human Snca transgenic mice**. Panels show the results obtained with the serum from patient 08–41, an asymptomatic LRRK2 patient. **(A)** Immunoblot analysis at 1/100 dilution against recombinant His-tag 1–60, His-tag 61–140, full length, and 1–108 (Stop 109) Snca showing that the main epitope was located at the N-terminal region of Snca (a.a. 1–60). **(B)** Immunoblot analysis of total serum upper part (08–411, 1/100 dilution) and affinity-purified anti-Snca antibodies [08–411 affinity-purified (A. P.) corresponding to 1/500 dilution of the original sample] against brain extracts prepared from wt, Snca knock-out, and human TH-Snca transgenic mice (Tg+).

To further characterize the presence of antibodies against Snca, we performed affinity-purification of the specific antibodies using human recombinant Snca. The eluted antibodies were tested by immunoblot against 1 μg of human recombinant Snca and all were positive, as expected. If those eluted antibodies have enough specificity and affinity for Snca, there should be able to detect Snca in crude brain extracts from wt mice and show no reactivity with brain extracts obtained from Snca null mice. In fact, as shown in Figure 4, patient 08–411, a non-manifesting R1441G LRRK2 carrier, had antibodies whose main epitope was located at the N-terminal region (a.a. 1–60) of Snca (Figure 4A). Both the original diluted plasma and the affinity-purified anti-Snca antibodies were able to detect Snca in the extracts of brain from wt and hSnca TH-driven transgenic mice, and the protein band detected was not found in brain extracts from Snca null mice (Figure 4B). These stringent test conditions were not met by any other of the original plasma/sera or affinity-purified antibodies, whose reaction with brain extracts was not significantly different from background reaction obtained with human plasma/sera that were negative for anti-Snca antibodies. These results clearly pointed out that most part of the human anti-Snca antibodies detected in patients and healthy controls are low affinity antibodies.

## Discussion

At the beginning of this study, we used recombinant purified Snca obtained as described previously (Martin-Clemente et al., 2004; Alvarez-Castelao and Castano, 2011) that was 95% homogenous by Coomassie blue staining. This protein preparation gave consistent results by ELISA, but when we checked some of the ELISA positive samples by immunoblot, we found that some of those clearly positive by ELISA did not recognize the Snca protein itself, but minor, likely *E. coli*, protein contaminants that were not obvious by Coomassie blue staining. These inconsistent results made us to introduce a further step of purification of Snca by RP-HPLC, rending a preparation of Snca close to 98% homogenous. The RP-HPLC Snca preparation gave consistent results by both techniques ELISA and immunoblot. Accordingly, we adopt the RP-HPLC step of Snca purification for all the studies reported here. The results we have obtained on the prevalence of anti-Snca antibodies in our case (iPD) and control (healthy subjects) study are in agreement with those published previously (Woulfe et al., 2002; Papachroni et al., 2007; Smith et al., 2012; Besong-Agbo et al., 2013) showing that there is no significant difference in the prevalence of anti-Snca antibodies between iPD patients and healthy controls. Furthermore, neither there is a higher prevalence of anti-Snca antibodies in non-manifesting (Asymp) or manifesting (Symp) LRRK2 carriers than in iPD patients or healthy controls when cut-off for anti-Snca positive reaction was established at OD ≥ 0.3 by endpoint ELISA and titer by immunoblot ≥1/100. When more stringent cut-off conditions were used for categorization of positive reactivity (OD ≥ 0.5 by endpoint ELISA and titer by immunoblot ≥1/200), significant difference in the prevalence of anti-Snca antibodies was found between non-manifesting (Asymp) LRRK2 patients and iPD patients or healthy controls. Under those restricted categorization of positive reactivity, we have also shown that anti-Snca antibodies are not significantly associated with other neurological or autoimmune diseases (see Table S1 in Supplementary Material), indicating some degree of disease specificity.

The anti-Snca antibodies detected were of the IgG class indicating that they are clearly distinct from the natural occurring and self-reactive autoantibodies commonly found in humans and animals, even in mice reared under germ-free condition, as they are mainly of the IgM class and polyreactive (Mannoor et al., 2013). Nevertheless, most of the anti-Snca IgG antibodies detected have low affinity for the antigen, as they were able to detect 1 μg of recombinant Snca, but unable to detect specifically the amount of Snca present in crude mouse brain extract (approximately 50 ng of Snca is present in 50 μg of total brain protein), exception made for one of the positive non-manifesting LRRK2 carriers (see Figure 3).

In spite of the low affinity of human anti-Snca antibodies, we have been able to clearly map the epitopes recognized by the human circulating antibodies using recombinant Snca protein constructs. It is noteworthy the absence of anti-N-terminal (a.a. 1–60) antibodies in healthy controls and the predominance of anti-N and C-terminal antibodies in LRRK2 carriers and iPD patients. We have also found clustering of anti-Snca reactivity within families for individuals carrying the LRRK2 mutation. Actually, 5 out of the 13 positive anti-Snca individuals that are LRRK2 carriers belong to family 1 (Table 2) and 1 of the healthy controls that had anti-Snca antibodies was a non-manifesting, non-carrier member of family 1 that has also anti-C-terminal (a.a. 109–140) anti-Snca antibodies. The main epitope of those anti-C-terminal antibodies from LRRK2 carriers seems to be located around amino acid N122. Unfortunately, we do not have samples from other family members of iPD patients or healthy controls (not typically considered in case-control studies); as a consequence, we could not check family clustering in those groups. These results extend a previous report that describes the presence of anti-Snca antibodies in familial, while genetically uncharacterized, forms of PD (Papachroni et al., 2007). Taken together, the interpretation of these results clearly point to a possible genetic (or common environmental) component for the formation of anti-Snca antibodies that may also affect the processing and/or presentation of Snca derived peptides to immune competent cells.

We have also tried to delineate if anti-Snca reactivity may have characteristics of an autoimmune response, as several autoimmune diseases (or only the presence of autoantibodies) also show familial aggregation where more than one member of a nuclear family has a single autoimmune disease (Cardenas-Roldan et al., 2013). Changes in antibody titer, appearance of epitope spreading, and/or conversion from negative to positive reactivity (or the opposite) along with time in different individuals would be indications of an “on-going” active immune response that could be due to an autoimmune (loss of tolerance) mechanism. We have limited follow-up samples of the patients carrying the LRRK2 mutation, and no follow-up samples of iPD patients or healthy controls. In the three cases of LRRK2 mutation carriers that we had samples 2 years apart, no difference was found in antibody titer or epitope mapping of the anti-Snca antibodies. Accordingly, in those analyzed patients the anti-Snca reactivity seems to be stable. None of the asymptomatic LRRK2 carriers have been converted to symptomatic along the course of this study. Clearly, further studies are required to ascertain the changes over time of the anti-Snca antibody response and repertoire to be able to ascertain a correlation of the immune response with PD pathology.

An interesting question is whether the anti-Snca antibody response is either beneficial or detrimental from the point of view of onset or progression of PD pathology. Studies in mice have reported that active immunization with Snca of mice transgenic for human Snca alleviates the Snca aggregation (Masliah et al., 2005) being more effective those antibodies directed against the C-terminal portion of Snca. More recent studies have shown that passive immunization of those transgenic mice with anti-C-terminal Snca monoclonal antibodies also alleviates behavioral and neuropathological deficits of the transgenic mice model (Masliah et al., 2011). Taking into account those results, it could be predicted that human subjects with anti-C-terminal Snca antibodies would be somehow “protected” in comparison to those individuals whose anti-Snca antibodies main epitope was located in other regions of Snca or have no antibodies. With the limited number of patients found in this study to have anti-C-terminal antibodies (11 individuals from a total of 21 positive) no such “protection” was evident by analyzing the possible correlation with different parameters including age, sex, years of disease evolution, or the disability score of PD (H&Y or the UPDRS). Furthermore, anti-C-terminal antibodies are the main class present in healthy control subjects, and as a consequence they may reflect a lost of self-tolerance as a part of the development of an autoimmune mechanism totally unrelated to PD pathology. Clearly, more extensive number of patients and healthy controls are needed to clarify the role of anti-Snca antibodies.

The presence of circulating anti-Snca antibodies has a clear tendency to cluster in certain families carrying the LRRK2 mutation, indicating that a genetic (or common environmental) factor could be implicated in the production of those antibodies in human subjects. Epitope mapping has demonstrated the absence of anti-N-terminal (a.a. 1–60) anti-Snca antibodies in positive healthy controls and the predominance of anti-N and C-terminal antibodies in LRRK2 mutation carriers and iPD patients, being N122 a key residue for recognition by the C-terminal directed human anti-Snca antibodies. Further familial cohort studies, not simple case-control studies, may allow ascertaining the possible existence of an anti-Snca (auto) immune response in humans and clarifying if there is some relationship between the anti-Snca reactivity and PD onset or progression. Finally, this type of studies may also provide some direct basis for the use of an immunological intervention for the treatment of PD and other synucleinopathies.

## Conflict of Interest Statement

The authors declare that the research was conducted in the absence of any commercial or financial relationships that could be construed as a potential conflict of interest.

## Supplementary Material

The Supplementary Material for this article can be found online at http://www.frontiersin.org/Journal/10.3389/fnagi.2014.00169/abstract
