# Supplementary material for: Epitope Mapping of Antibodies to Alpha-Synuclein in LRRK2 Mutation Carriers, Idiopathic Parkinson Disease Patients, and Healthy Controls
Source: Front Aging Neurosci. 2014 Jul 15;6:169. doi: 10.3389/fnagi.2014.00169 (PMC4097207; doi:10.3389/fnagi.2014.00169)
Supplement: Supplementary file 1 [file Presentation1.PDF]

Supplementary Tables

Supplementary Table 1. Demographic data and prevalence of anti-Snca antibodies in patients with different neurological diseases, autoimmune diseases and healthy controls from another geographical region (Madrid, Spain).

Condition	Age (mean ± SD)	Sex (Male/Female)	Total	Positive anti-Snca antibodies
Alzheimer's disease	74.5 ± 5.9	20/23	43	0
Progressive Supranuclear Palsy	76.2± 7.7	8/9	17	0
Multiple Sclerosis	42 ± 9	20/25	45	1
Primary Biliary Cirrhosis	75 ± 10	12/24	36	0
Systemic lupus erythematosus	60 ± 13	1/24	25	0
Sjogren syndrome	66 ± 14	0/15	15	0
Healthy subjects	72 ± 9	19/20	39	1

Supplementary Table 2. Determination of accuracy of Snca antibodies determination by ELISA assay.

Samples	Expected	Observed	% Recovery
1	0.74	0.80	108.1
2	0.55	0.56	101.8
3	0.30	0.28	93.3
4	0.21	0.20	95.2
5	0.14	0.13	92.8

Plasma from five different patients with different levels of reactivity was diluted 1:2 with a negative sample and assayed in triplicate. The expected value was estimated as

half of the value obtained with the undiluted sample, accuracy was estimated as percent (expected/obtained values x100).

Supplementary Table 3. Intra-assay precision (within-run) of anti-Snca antibodies determination by ELISA assay.

Samples n=10	Average O.D. 405 nm	S. D.	CV (%)
1	1.42	0.04	2.81
2	1.28	0.05	3.9
3	0.59	0.05	8.47
4	0.46	0.04	8.69
5	0.26	0.03	11.53
6	0.15	0.02	13.3

Plasma from six different patients with different levels of reactivity was run 10 times and the average and CV was calculated.

Supplementary Table 4. Inter-assay precision of anti- Snca antibodies determination by ELISA assay.

Samples	Average O.D. 405 nm	S. D.	CV (%)
1	1.54	0.10	6.49
2	1.30	0.10	7.60
3	0.58	0.05	8.62
4	0.49	0.03	6.12
5	0.27	0.03	11.1
6	0.15	0.02	13.3

Plasma from six different patients with different levels of reactivity was run by triplicate in two different assays performed one week apart and the average and CV was calculated.
